# 801. Patient Reported Experience with Influenza Episode and Impact on Life and Work

**DOI:** 10.1093/ofid/ofab466.997

**Published:** 2021-12-04

**Authors:** Nate Way, Ashley Martin, Chris Wallick, Edward Neuberger, Mitra Corral

**Affiliations:** 1 Health Outcomes Practice, Kantar Health, San Mateo, CA; 2 Genentech, Inc., South San Francisco, CA

## Abstract

**Background:**

Influenza is a highly prevalent seasonal disease that has a wide range of impact on patients, including experiencing symptoms, social isolation, missing work, and worrying about transmitting to others. The aim of this study was to better understand patients’ experience with influenza, areas of life that were most impacted and what matters most to patients.

**Methods:**

Data for this study were obtained from two online quantitative surveys of influenza patients: A pool of respondents who previously completed the National Health and Wellness Survey (NHWS) (N=74,977) OR from Lightspeed M3 Global’s online “General Panel” (N=500,000+) in the US between January 2020 through May 2020. A total sample of 1,005 patients >18 years of age and having a self-reported diagnosis of influenza by a healthcare professional within the last 90 days were included. Outcomes related to patient demographics, health-related characteristics, perspectives on the influenza episode and productivity impairment (measured by the Work Productivity and Activity Impairment questionnaire) were collected.

**Results:**

Influenza patients reported greatest impact on physical activity, running errands and chores outside the home, and overall lifestyle. 64% were employed at the time they experienced influenza. They missed a mean 23 hours of work due to problems associated with their influenza and actually worked for 15 hours during the 7 days after first experiencing symptoms. Mean absenteeism (work time missed), presenteeism (impairment at work), overall work impairment, and activity impairment was 59%, 73%, 87% and 75%, respectively. For employed respondents, 60% missed work because of transmission worry; about 40% of patients had somebody else get flu shortly after them and about 68% of those people felt responsible for transmitting.

**Conclusion:**

Influenza greatly reduced respondents’ ability to be productive at work and in their daily activities outside of work. Worry about transmitting an influenza infection to others was a highly influential motivator for missed work or other responsibilities.

**Disclosures:**

**Nate Way, PhD**, **Genentech, Inc.** (Grant/Research Support)**Kantar Health** (Employee) **Ashley Martin, PhD**, **Genentech, Inc.** (Grant/Research Support)**Kantar Health** (Employee) **Chris Wallick, PharmD, MS**, **Genentech, Inc.** (Employee, Shareholder) **Edward Neuberger, PharmD, MBA, MS**, **Genentech, Inc.** (Employee, Shareholder) **Mitra Corral, MS, MPH**, **Genentech, Inc.** (Employee, Shareholder)

